# Catamenial pneumothorax: A case report

**DOI:** 10.1016/j.radcr.2025.04.033

**Published:** 2025-05-08

**Authors:** Luong Ngoc Trung, Viet Huan Le, Pham Nguyen Thanh Nam, Tran Minh Bao Luan

**Affiliations:** a Department of Thoracic, Vascular and Endovascular Surgery, FV Hospital, Ho Chi Minh City 70000, Vietnam; b Department of Cardiovascular – Thoracic Surgery, University of Medicine and Pharmacy of Ho Chi Minh City, Ho Chi Minh City, 70000, Vietnam; c Department of Thoracic and Vascular Surgery, University Medical Center, Ho Chi Minh City, 70000, Vietnam; d Department of Thoracic Surgery, Khanh Hoa General Hospital, Nha Trang City, Khanh Hoa Province, 65000, Vietnam

**Keywords:** Catamenial pneumothorax, Thoracic endometriosis, Diaphragmatic defects

## Abstract

Catamenial pneumothorax is defined as spontaneous, recurrent pneumothorax occurring in women of reproductive age and associated with the menstrual cycle. This condition is rare and challenging to diagnose, despite increasing awareness due to case and series reports in recent years. Here, we present a clinical case of a 47-year-old woman with a history of recurrent right-sided pneumothorax requiring chest drainage multiple times from 2016 to 2022. Her pneumothorax episodes were cyclical, varying in severity. The diagnosis of catamenial pneumothorax was established, and she underwent thoracoscopic surgery to repair the lesions. One year postoperatively, there has been no recurrence of pneumothorax.

## Introduction

Catamenial pneumothorax (CP) is a spontaneous, recurrent pneumothorax that occurs in women of reproductive age in association with the menstrual cycle, excluding those with underlying lung disease [1]. Symptoms often occur at different times in relation to menstruation: before, during, or after with a variable duration ranging from hours to up to 7 days [[2], [3], [4]]. CP is the most common manifestation of thoracic endometriosis syndrome, which includes hemothorax, hemoptysis, pulmonary nodules, and pneumothorax. Although it accounts for only 3%-6% of spontaneous pneumothorax cases, CP often remains underdiagnosed. Approximately 87.5%-100% of cases occur on the right side but can occasionally be bilateral or affect the left side [2]. First described by Maurer in 1958 [5], the term “catamenial pneumothorax” was introduced by Lillington in 1972 [6]. Although our understanding of CP has improved, precise diagnosis remains a clinical challenge [7].

## Case report

**Fig. 1:**
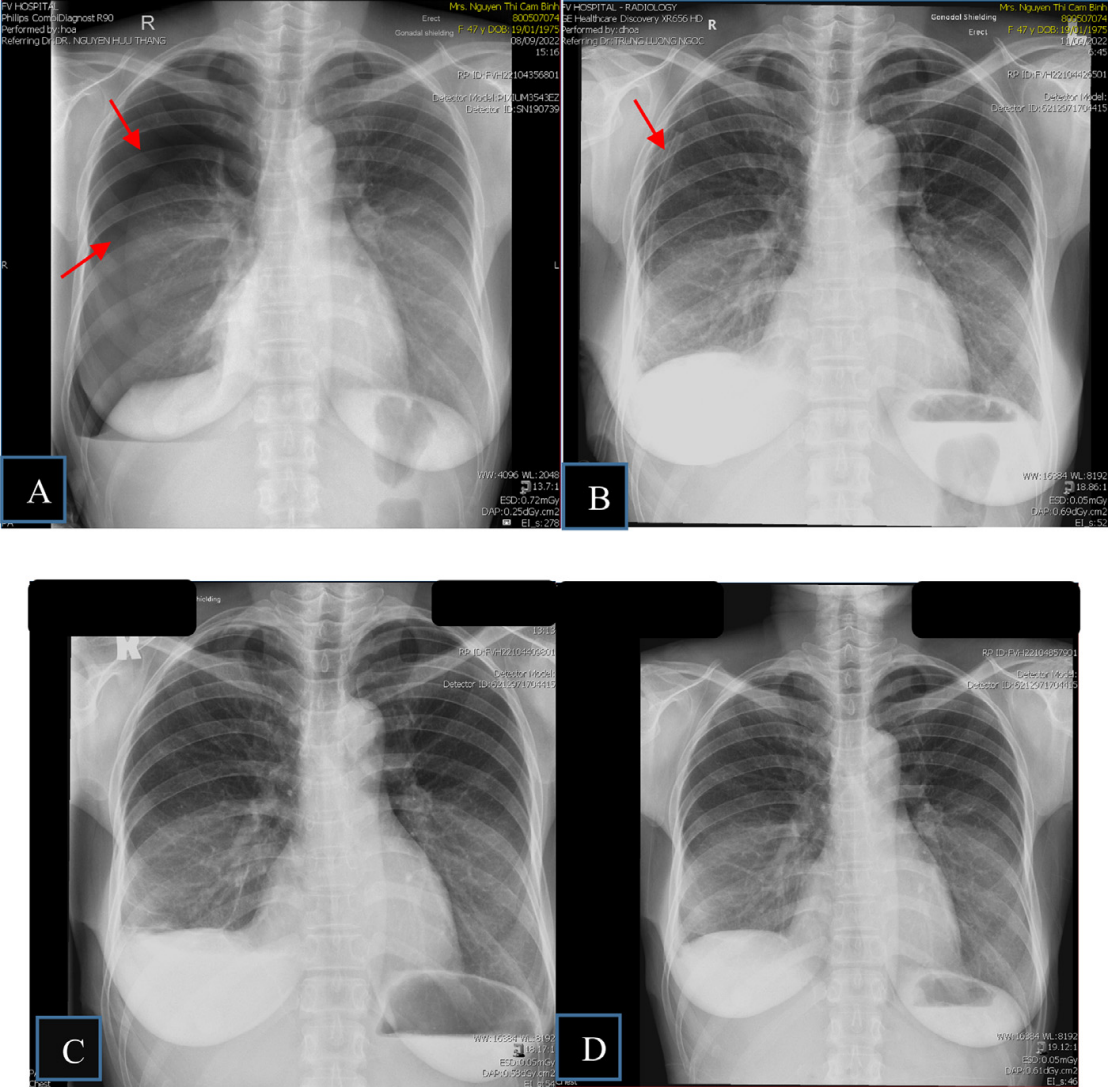
Chest X-ray results. (A) X-ray on September 8, 2022: Significant right-sided pneumothorax. (B) X-ray on September 11, 2022: Right-sided pleural drainage tube. (C) X-ray on September 20, 2022: No pneumothorax detected. (D) X-ray on September 29, 2022: No pneumothorax detected.

**Fig. 2:**
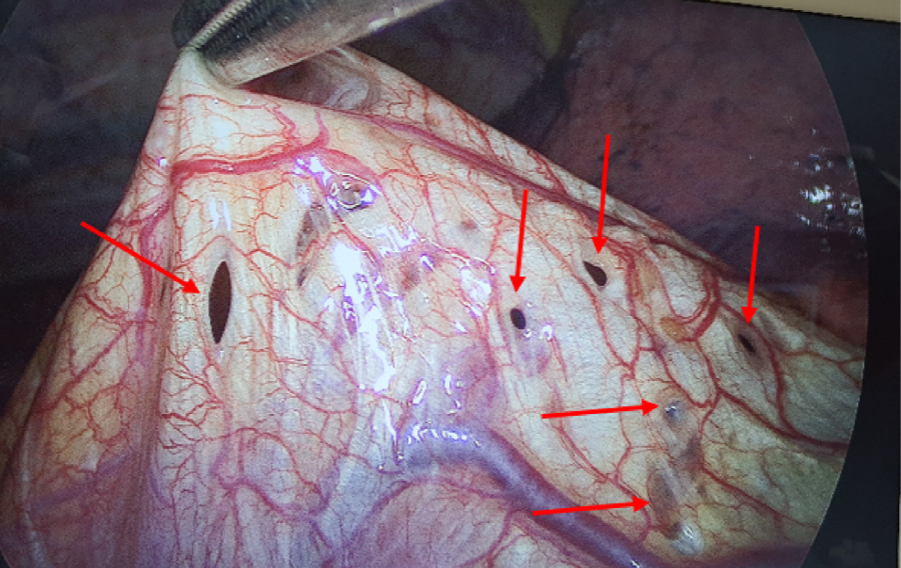
Lesions detected on the diaphragmatic surface.

**Fig. 3:**
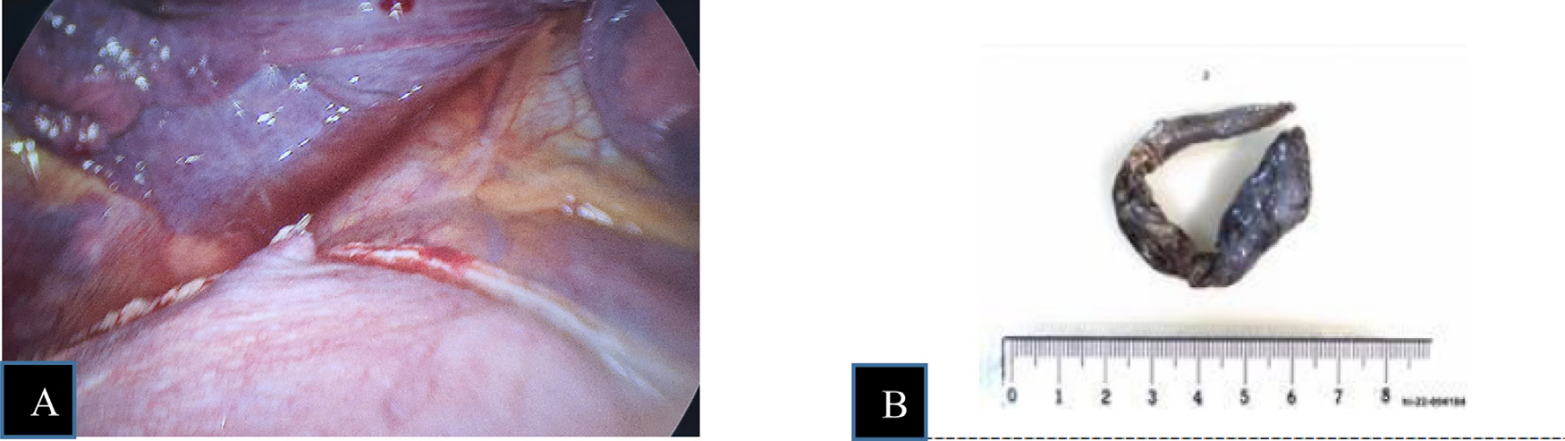
Image of right diaphragm repair and suturing. (A) Right diaphragm repair and suturing. (B) A suspected diaphragmatic lesion was excised for pathology.

**Fig. 4:**
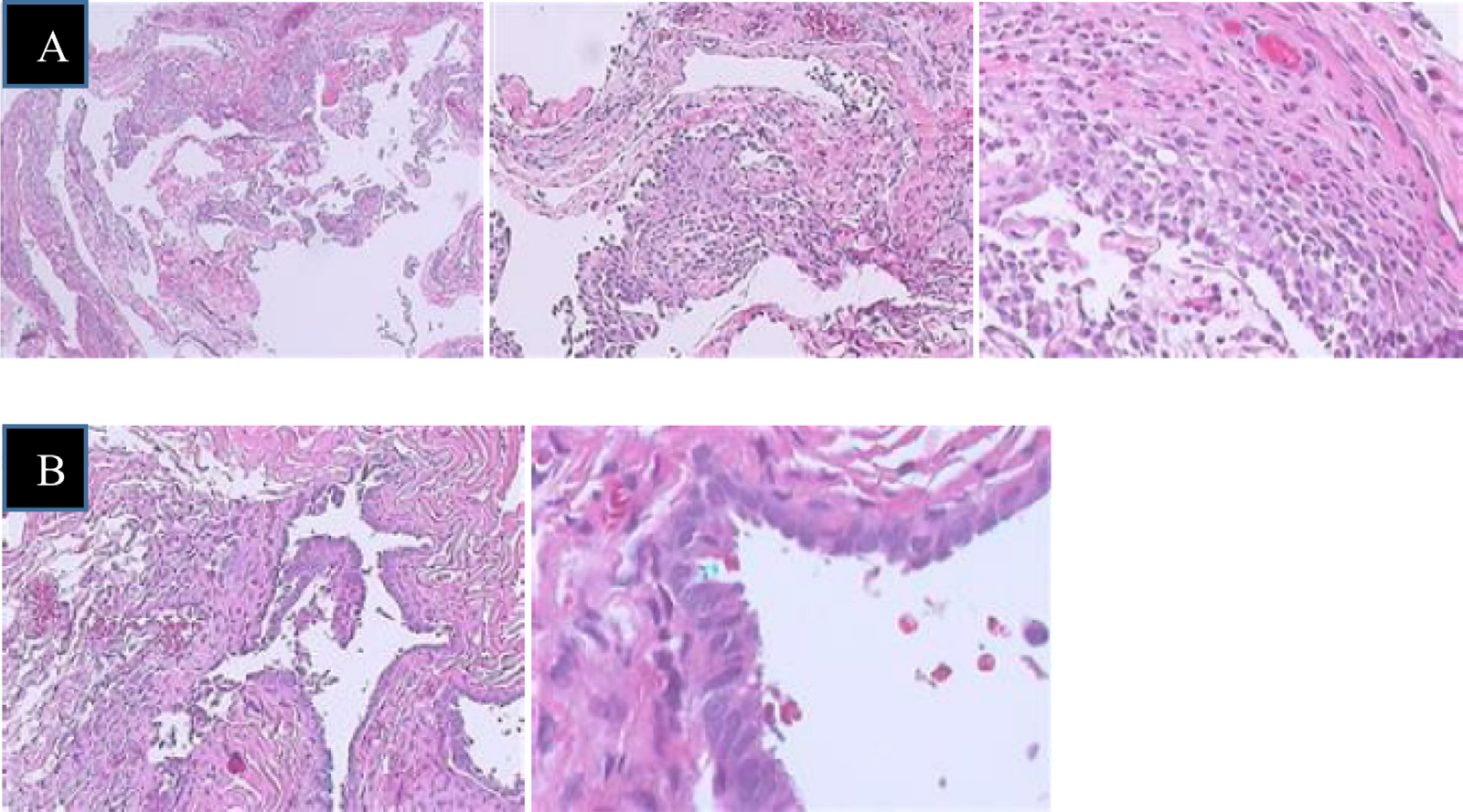
Histopathological image. (A) Labeled as “Endometriosis of right pleural cavity”: Sections show papillary serosal surface lined by mesothelial cells and covered focally by aggregates of mixed inflammatory cells mainly macrophages and smaller amount of eosinophils and neutrophils. Fibrous stroma is infiltrated focally by lymphocytes. No tissue suspicious for Endometriotic tissue is seen. Hemosiderin pigment is not observed. (B) Right diaphragm: The diaphragm show focal glandular structures that could not excluded endometrial glands. Endometrial stroma is not obvious.

A 47-year-old female office worker presented at 2:20 PM on September 8, 2022, with complaints of cough, chest tightness, and dyspnea. The patient's family history is unremarkable. Her history revealed 6 episodes of right-sided chest drainage over 9 years at different medical centers. Five episodes occurred during the first 3 years, with subsequent recurrences left untreated by the patient. Chest X-rays revealed moderate right-sided pneumothorax (Fig. 1A), which worsened over 2 days as symptoms intensified. On admission, she was hemodynamically stable with an oxygen saturation of 97% on room air. Physical examination showed diminished breath sounds over the right lung. X-ray confirmed a large recurrent pneumothorax. This patient did not have bullae detected on previous imaging, making primary spontaneous pneumothorax due to bullae rupture unlikely. Secondary spontaneous pneumothorax was also ruled out, as the patient had no history of chronic lung disease. Additionally, there was no history of trauma or malignancy, allowing us to exclude other potential causes of pneumothorax. After a thorough diagnostic process, other possible causes were eliminated, as the patient had experienced previous episodes of pneumothorax occurring in relation to the menstrual cycle, and the final diagnosis was recurrent pneumothorax associated with menstruation (CP), linked to diaphragmatic and pleural endometriosis. Given this diagnosis, the patient underwent video-assisted thoracoscopic surgery (VATS), during which multiple fenestrations resembling a sieve were observed on the right diaphragm (Fig. 2). Pale yellow nodules adhered to the diaphragmatic surface and parietal pleura. No bullae or air leaks were noted in the lung tissue. The diaphragmatic defects were repaired using a stapler (endoGIA 4.5/60 mm) (Fig. 3), mechanical pleurodesis with talc was performed, and nodular lesions were biopsied. Postoperatively, the pneumothorax resolved, and the chest tube was removed after 2 days. Follow-ups at one week, one month, 3 months, and one year showed clinical stability without recurrence. Histopathological examination revealed 2 specimens. I: A 0.6 × 0.4 × 0.2 cm irregular gray-white tissue, labeled as “endometriosis of right pleural cavity,” showed papillary mesothelial hyperplasia, chronic inflammation, and no endometriotic tissue or hemosiderin pigment. II: A 9.5 × 2 × 1.4 cm wedge-shaped lung tissue, labeled as “right diaphragm resection,” revealed focal glandular structures suggesting endometrial glands, although stroma was not obvious (Fig. 4).

## Discussion

CP is defined as spontaneous, recurrent pneumothorax occurring at least twice in temporal association with menstruation [8]. Endometriosis is a condition in which uterine tissue is abnormally located outside the uterine cavity. It can be localized within the pelvic region or outside the pelvis, and its name may vary depending on the location of the ectopic tissue. Thoracic endometriosis refers to ectopic endometrial tissue in the thoracic cavity and is the most common extrapelvic manifestation of endometriosis.

First described in 1958 by Maurer [5], the term “catamenial pneumothorax” was introduced in 1972 by Lillington [6]. In 1996, Joseph and colleagues coined the term “thoracic endometriosis syndrome” to describe the clinical condition of thoracic endometriosis, encompassing various symptoms such as pneumothorax, hemothorax, hemoptysis, and pulmonary nodular lesions. The prevalence of endometriosis is higher among Asians compared to Europeans (15% vs 5%-10%) [9]. According to the literature, ectopic lesions can be found in the lungs, pleura, and diaphragmatic surfaces [9,10]. Spontaneous pneumothorax is the most common symptom of thoracic endometriosis syndrome (72%-73%) [2,4]. However, endometrial tissue is not present in all patients with pneumothorax, being detected in only about 52.1%, with 32% of cases showing lesions on the diaphragmatic surface [11].

Women of reproductive age presenting with cyclical symptoms such as dysuria, indigestion, hematuria, or rectal bleeding; recurrent pneumothorax, hemothorax, hemoptysis, chest pain, and fatigue should be considered for endometriosis. However, clinical symptoms alone are insufficient for diagnosis [8]. Currently, there are no clear diagnostic criteria for a definitive diagnosis, and the most crucial factor is the medical history, including recurrent symptoms occurring in sync with the menstrual cycle. There are no specific imaging standards [2,12], A standard chest X-ray is often recommended [2] and may reveal pneumothorax. CT scans and MRIs are less commonly indicated [2], but may detect diaphragmatic defects described as “air-filled bubbles”[12], In some cases, MRI can aid in diagnosing endometriosis and identifying thoracic lesions [2,12] . Observations during surgery, such as diaphragmatic defects or pleural lesions, serve as diagnostic clues.

There are several theories explaining the mechanism behind catamenial pneumothorax (CP), each highlighting different pathways through which air may enter the pleural cavity or how endometrial tissue might contribute to lung complications: [2].

The transgenital-transdiaphragmatic passage of air theory suggests that air from the atmosphere can enter the uterus through the cervix, particularly during menstruation when cervical mucus is absent. This air then moves through the fallopian tubes into the peritoneal cavity and subsequently reaches the pleural space via small pre-existing openings in the diaphragm or defects that develop over time. This theory proposes a direct connection between the reproductive system and the pleural cavity, allowing air to pass upwards and cause recurrent pneumothorax.

The migration theory focuses on the role of endometrial tissue in CP. It proposes that during retrograde menstruation, endometrial cells travel backward into the pelvic cavity and, over time, migrate toward the diaphragm via peritoneal fluid movement. These ectopic endometrial implants undergo cyclical necrosis, weakening the diaphragm and creating perforations. Through these openings, endometrial cells can enter the pleural cavity and attach to the lung's surface. During each menstrual cycle, the repeated breakdown of these ectopic tissues may lead to alveolar rupture, ultimately causing pneumothorax.

The prostaglandin theory offers an alternative explanation for CP in cases where thoracic endometriosis is not detected. It suggests that hormonal fluctuations during the menstrual cycle influence the permeability of blood vessels, alter diaphragmatic function, and facilitate the entry of air into the pleural cavity. Additionally, prostaglandin F2α, a compound involved in uterine contractions, may trigger bronchoconstriction and pulmonary vasospasm, leading to alveolar air leakage. Increased intra-abdominal pressure during menstruation might also force air through microscopic defects in the diaphragm. This theory emphasizes the impact of hormonal and physiological changes rather than the presence of ectopic endometrial tissue.

The first-line treatment is thoracoscopic surgery to remove thoracic lesions, reconstruct anatomical structures, and perform pleurodesis [9,11,12]. However, if the patient declines surgery or is not a suitable candidate, hormonal therapy may be considered [8,9]. In cases where the patient opts against thoracic surgery and has no plans for future childbearing, bilateral oophorectomy may also be considered [8].

Medical treatment involves the use of GnRH hormones for 6-12 months. The goal is to suppress the activity of ectopic endometrial tissue while allowing complete pleural adhesion [8,13]. Additionally, other treatment options include contraceptives, danazol, and progestins [9,12].

As this is a rare cause of spontaneous pneumothorax, patients often present to thoracic surgeons first with acute symptoms of pneumothorax. To optimize treatment, in addition to thoracoscopic surgery, hormonal therapy under the supervision of a gynecologist is essential. Therefore, collaboration between thoracic surgeons and gynecologists is crucial for the optimal diagnosis and management of the patient [8,13].

## Conclusion

Catamenial pneumothorax is a rare and often overlooked cause of spontaneous pneumothorax, associated with thoracic endometriosis. Accurate diagnosis and definitive treatment can prevent recurrence, reduce treatment costs, and improve patients' quality of life. The first-line treatment remains thoracoscopic surgery, with adjunctive medical therapy as an option to achieve optimal outcomes. Therefore, collaboration between thoracic surgeons and gynecologists is essential.

## Patient consent

Written informed consent was obtained from the patient for the publication of this case report and accompanying images
